# Biofortified Rice Provides Rich Sakuranetin in Endosperm

**DOI:** 10.1186/s12284-024-00697-w

**Published:** 2024-03-02

**Authors:** Yao Zhao, Jitao Hu, Zhongjing Zhou, Linying Li, Xueying Zhang, Yuqing He, Chi Zhang, Junmin Wang, Gaojie Hong

**Affiliations:** 1 State Key Laboratory for Managing Biotic and Chemical Treats to the Quality and Safety of Agro-Products, Key Laboratory of Biotechnology in Plant Protection of MOA of China and Zhejiang Province, Institute of Virology and Biotechnology, Zhejiang Academy of Agricultural Sciences, Hangzhou, 310021 Zhejiang China; 2 Central Laboratory, State Key Laboratory for Managing Biotic and Chemical Threats to the Quality and Safety of Agro-Products, Zhejiang Academy of Agricultural Sciences, Hangzhou, 310021 Zhejiang China; 3 Institute of Crops and Nuclear Technology Utilization, Zhejiang Academy of Agricultural Sciences, Hangzhou, 310021 Zhejiang China

**Keywords:** Biofortified rice, Sakuranetin biosynthesis, Metabolic engineering, MALDI-MS imaging, Rice endosperm

## Abstract

Sakuranetin plays a key role as a phytoalexin in plant resistance to biotic and abiotic stresses, and possesses diverse health-promoting benefits. However, mature rice seeds do not contain detectable levels of sakuranetin. In the present study, a transgenic rice plant was developed in which the promoter of an endosperm-specific glutelin gene *OsGluD-1* drives the expression of a specific enzyme naringenin 7-*O*-methyltransferase (NOMT) for sakuranetin biosynthesis. The presence of naringenin, which serves as the biosynthetic precursor of sakuranetin made this modification feasible in theory. Liquid chromatography tandem mass spectrometry (LC–MS/MS) validated that the seeds of transgenic rice accumulated remarkable sakuranetin at the mature stage, and higher at the filling stage. In addition, the panicle blast resistance of transgenic rice was significantly higher than that of the wild type. Specially, the matrix-assisted laser desorption/ionization mass spectrometry (MALDI-MS) imaging was performed to detect the content and spatial distribution of sakuranetin and other nutritional metabolites in transgenic rice seeds. Notably, this genetic modification also did not change the nutritional and quality indicators such as soluble sugars, total amino acids, total flavonoids, amylose, total protein, and free amino acid content in rice. Meanwhile, the phenotypes of the transgenic plant during the whole growth and developmental periods and agricultural traits such as grain width, grain length, and 1000-grain weight exhibited no significant differences from the wild type. Collectively, the study provides a conceptual advance on cultivating sakuranetin-rich biofortified rice by metabolic engineering. This new breeding idea may not only enhance the disease resistance of cereal crop seeds but also improve the nutritional value of grains for human health benefits.

**Supplementary Information:**

The online version contains supplementary material available at 10.1186/s12284-024-00697-w.

## Introduction

**Fig. 1:**
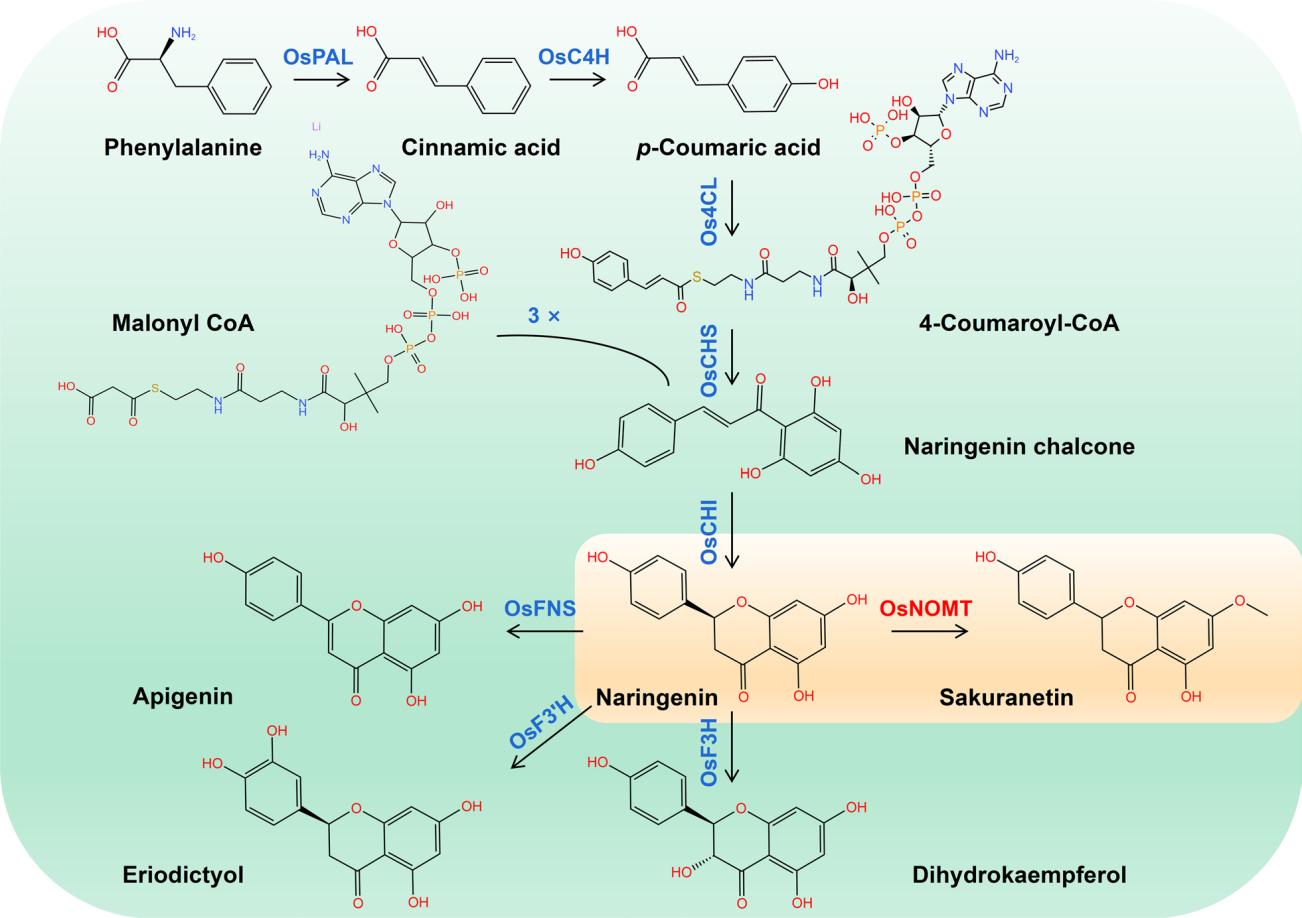
The pathways of sakuranetin biosynthesis in rice. Metabolites are indicated in black and different enzymes are indicated in color. PAL, phenylalanine ammonialyase; C4H, cinnamic acid 4-hydrolase; 4CL, coumarin-CoA ligase; CHS, chalcone synthase; CHI, chalcone isomerase; FNS, flavone synthase; F3'H, flavonoid-3'-hydroxylase; F3H, flavanone-3-hydroxylase; NOMT, Naringenin 7-*O*-methyltransferase

Sakuranetin (4', 5-dihydroxy-7-methoxyflavanone) is an inducible secondary metabolite, that belongs to the class of flavonoids known as flavanones. It was first extracted from the bark of Chinese cherries (*Prunus pseudocerasus*), and subsequently identified in a series of rice studies as a new phytoalexin with brilliant activity against rice blast (Kodama et al. 1992; Hasegawa et al. 2014; Murata et al. 2020). In addition to the accumulation in rice in response to *Magnaporthe oryzae* infection, sakuranetin also possesses broad-spectrum resistance to a wide range of phytopathogenic fungi (e.g., *Rhizoctonia solani* and *Bipolaris oryzae*) and bacteria (e.g., *Xanthomonas oryzae* pv. *oryzae*, *Xanthomonas oryzae* pv. *oryzicola*, and *Burkholderia glumae*) (Hasegawa et al. 2014; Park et al. 2014). Moreover, UV radiation, CuCl_2_, jasmonic acid, brassinosteroid, and brown planthopper (BPH) attack also induced the accumulation of sakuranetin in rice (Shimizu et al. 2012b; Ogawa et al. 2017; Liu et al. 2023; He et al. 2024). In fact, beyond acting as a phytoalexin in plants, sakuranetin holds tremendous promise as a nutraceutical or pharmaceutical agent, as well as an active ingredient in skincare products, due to its exceptional antiviral, anticancer, anti-inflammatory, antiparasitic, antioxidant, and anti-allergic properties (Melo et al. 2018; Stompor 2020; Junaid et al. 2022). For example, sakuranetin is effective against rhinoviruses, influenza B, B16BL6 melanoma, ESCC (esophageal squamous cell carcinoma), Colo 320 (colon cancer), leishmaniasis, and Chagas disease (Ugocsai et al. 2005; Hong and Ying 2015; Drira and Sakamoto 2016; Choi 2017; Kwon et al. 2018; Santana et al. 2019). The biosynthetic pathway of sakuranetin in rice has been clearly studied, as illustrated in Fig. 1, which is synthesized through the flavonoid branch of the general phenylpropanoid pathway. Enzymes commonly found in flavonoid metabolic pathways, including phenylalanine ammonialyase (PAL), cinnamic acid 4-hydrolase (C4H), coumarin-CoA ligase (4CL), chalcone synthase (CHS), and chalcone isomerase (CHI), are involved in the synthesis of sakuranetin in rice. Furthermore, the methyltransferase NOMT (Naringenin 7-*O*-methyltransferase) plays a crucial role as a specific key enzyme that catalyzes the biosynthesis of sakuranetin from the naringenin (Shimizu et al. 2012a; Rakwal et al. 2000). Even though the pathogen resistance function of sakuranin in rice leaves has been widely concerned, previous metabolomics found that sakuranetin was undetectable in mature rice seeds (Shi et al. 2022; Tang et al. 2022).

In recent years, many studies have used metabolic engineering and synthetic biology to modify flavonoid metabolic and regulatory pathways to generate biofortified crops. The overexpression of MYB transcription factors has been shown to enhance anthocyanin content significantly, resulting in the production of pigment-rich crops such as purple-fleshed sweet potato, purple cauliflower, red-fleshed apple, blood orange, and purple pummelo (Mano et al. 2007; Espley et al. 2009; Chiu et al. 2010; Butelli et al. 2012; Huang et al. 2018). Besides, driving a functional *SlAN2-like* gene through fruit-specific promoters also leads to the activation of the entire anthocyanin biosynthesis pathway in tomatoes and massive accumulation of anthocyanins in both the peel and flesh (Sun et al. 2020). In addition to being widely used in tissues such as fruits, leaves, and flowers of fruits and vegetables, metabolic engineering to modify flavonoid metabolic pathways has also been applied to rice endosperm. Flavonoids are absent or very low in the rice endosperm, although anthocyanins are abundant in the pericarp of natural black and red rice grains. Ogo et al. successfully produced a transgenic rice plant capable of synthesizing several classes of flavonoids in its seeds, which was accomplished by introducing multiple genes encoding enzymes involved in flavonoid synthesis, from phenylalanine to the target flavonoids (Ogo et al. 2013). Similarly, Zhu et al. produced “Purple Endosperm Rice” (called *Zijingmi* in Chinese) by accumulating anthocyanins in endosperm using a high-efficiency multigene vector system TGSII, and this binary vector was constructed containing two regulatory genes and six structural genes for endosperm-specific anthocyanin synthesis (Zhu et al. 2017).

As we all know, rice seeds not only provide energy for plant growth and development, but are also an essential source of various nutrients for humans. Remarkably, rice endosperm has emerged as an ideal bioreactor for plant molecular farming to produce and store the recombinant proteins (antigens and vaccines/edible vaccines, pharmaceutical proteins and peptides, and antibodies) and bioactive metabolites (vitamins and minerals, carotenoids, and flavonoids). This is primarily due to its advantages, including rich genetic and bioinformatic resources, strong expression of complex proteins, ease of extraction and processing, low production costs, and few biosafety risks (Zhu et al. 2022). For instance, the expression of daffodil or maize phytoene synthase gene *PSY* in the rice endosperm can produce different amounts of beta-carotene that are lacking in rice seeds, thus cultivating the famous “Golden Rice” rich in provitamin A (Ye et al. 2000; Paine et al. 2005). Zhu et al. used TGSII system led to de novo astaxanthin biosynthesis in rice endosperm by co-express *PSY*, *CrtI*, *BHY*, and *BKT* gene, producing astaxanthin-enriched “Astaxanthin Rice” (called *Chijingmi* in Chinese) (Zhu et al. 2018). Using the 2A-mediated polycistronic method, Ha et al. achieved the biosynthesis of zeaxanthin, astaxanthin, and capsanthin in rice endosperm by stepwise pathway engineering (Ha et al. 2019). However, the above modifications to rice endosperm are all from the nutrition and health perspective, without considering plant resistance. Sakuranetin, the sole flavonoid phytoalexin with favorable implications for human health, has the potential to produce nutritionally enhanced rice grains via metabolic engineering, while also conferring disease resistance.

Glutelins are the most abundant seed storage proteins (SSPs) in the rice endosperm (70–80%) (Kawakatsu et al. 2008). Glutelin gene *OsGluD-1* attains maximal expression levels in the starchy endosperm, commencing from 5 d after flowering (DAF) and increasing through 30 DAF (Kawakatsu et al. 2010). In this study, we employed the promoter of *OsGluD-1* to drive the expression of *OsNOMT*, generating biofortified rice with rich sakuranetin in the endosperm. Except for the conventional liquid chromatography tandem mass spectrometry (LC–MS/MS) technique, a nondestructive, labeling-free, in situ, and visual emerging means matrix-assisted laser desorption/ionization mass spectrometry (MALDI-MS) imaging was performed to detect the spatial distribution of sakuranetin and its biosynthetic pathway metabolites in *pOsGluD-1::OsNOMT* seeds. After observation and validation, the nutritional quality and growth of *pOsGluD-1::OsNOMT* were consistent with the wild type. Our work provides a successful example of biofortified crop cultivation through metabolic engineering, the biofortified rice not only enhances rice panicle blast resistance but can also be consumed as a health-promoting food and processed into dietary supplements.

## Materials and Methods

### Plant Material and Growth Conditions

The seeds of wild type (*Oryza sativa japonica* variety Zhonghua11) and transgenic lines of rice were soaked in tap water at 37 °C for 2–3 d to germination. The germinated seeds were planted in 8 × 12 cm^2^ boxes (individual 96-well plates) containing 1/2 MS culture solution (pH 5.8) and cultivated in a growth chamber at 30 °C, 60% relative humidity under a 12 h light/12 h dark photoperiod. Rice field in Zhejiang Academy of Agricultural Sciences (30° 18′ 44″ N, 120° 11′ 33″ E), Hangzhou, Zhejiang Province, China.

### Vector Construction and Genetic Transformation

For analysis of *pOsNOMT::GUS* expression patterns, the genomic fragment of the *OsNOMT* (Os12g0240900) promoter region starting 2.5 kb upstream of the ATG codon was amplified by PCR with the primer pair pOsNOMT-F/pOsNOMT-R and sequenced. The resulting fragment was inserted into *PstI* and *BamHI* cloning sites of the expression cassette of the pCAMBIA1300-GUSplus vector to generate the plasmid *pOsNOMT::GUS*. For constructing *p35S::OsNOMT-GFP* plasmid, the full-length coding sequences of *OsNOMT* were amplified via RT-PCR with the primer pair OsNOMT-CDS-F1/OsNOMT-CDS-R1 and cloned into the pCAMBIA1300-35S-eGFP vector at the *SpeI* site. To construct the *pGluD-1::OsNOMT* plasmid, the *OsNOMT* CDS was first amplified with the primer pair OsNOMT-CDS-F2/OsNOMT-CDS-R2 and cloned into the pCAMBIA1300 vector at the *PstI* and *HindIII* sites. Subsequently, the genomic fragment of the *OsGluD-1* (Os02g0249000) promoter region starting 1.6 kb upstream of the ATG codon was amplified by PCR with the primer pair pGluD-1-F/pGluD-1-R and inserted into the above construct at the *KpnI* and *PstI* sites. For generating the transgenic plants of rice, the desired constructs were transferred into *Agrobacterium tumefaciens* strain EHA105 as described previously (Toki et al. 2006). All transgenic plants were screened and identified by PCR amplification using universal primers (Hyg-F/Hyg-R) and the corresponding specific primers. The PCR primers can be seen in Additional file 1: Table S2.

### GUS Staining

The different tissues of *pOsNOMT::GUS* were harvested and immersed into the staining buffer (50 mM sodium phosphate, pH 7.0, 10 mM Na_2_EDTA, 0.5 mM potassium ferricyanide, 0.5 mM potassium ferrocyanide, 0.1% (v/v) Triton X-100, and 2 mM X-Gluc), vacuum-infiltrated for 5 min and incubated at 37 °C overnight. Ultimately, the tissues were cleared in 75% ethanol at 37 °C several times until the chlorophyll was completely removed. The staining patterns of GUS were observed and photographed with the digital microscope (Keyence, VHX-950F) or digital camera (Canon, 80D).

### Determination of Sakuranetin and Naringenin Content

To detect the content of sakuranetin and naringenin, the different tissues of rice were harvested and ground the sample with liquid nitrogen. The sample with fresh weight of 0.1 g was weighed, and added 1.6 mL extraction buffer (the volume ratio of ethanol: water: acetonitrile: acetic acid is 79: 13.99: 7: 0.01). After 30 min of 100 Hz ultrasound, it was rotated overnight at 4 ℃. Centrifuged at 13,000 rpm for 20 min, 1 mL supernatant was absorbed. Centrifuged at 13,000 rpm for 20 min for the second time, 700 μL supernatant was absorbed into mass spectrometry tube to detected sakuranetin and naringenin by LC–MS/MS (SCIEX Triple Quad 5500+ LC–MS/MS).

### RNA Extraction and qRT-PCR

Various rice tissues were frozen in liquid nitrogen and mixed with Trizol reagent. Total RNAs were extracted according to the manufacturer’s instructions (Invitrogen). cDNA was produced from 1 μg of total RNA using a reverse transcription system with gDNA Eraser (Vazyme). Quantitative real-time PCR was performed with SYBR Premix Ex TaqII (Takara) using an ABI7900HT Sequence Detection System and analyzed using the 2^–△△CT^ method. The *OsACTIN1* (Os03g0718100) was used as an internal reference to normalize the gene expression data. Three biological and two technical repeats were performed in the experiments. The qPCR primer pairs for gene amplification are given in Additional file 1: Table S2.

### Sample Preparation and MALDI-MS Imaging

Rice seeds at 25 DAF were harvested and husked. The seeds were then embedded with 2% CMC in the embedding box and frozen in a dry ice-ethanol bath. After the embedding medium has fully solidified and turned white, the box can be taken out and stored at − 80 °C. Before the section, samples were placed in a − 20 °C refrigerator to equilibrate for 1 h and fixed in three drops of distilled water for cutting. The seeds were sectioned at 20 μm thickness using a Leica CM1950 cryostat (Leica Microsystems GmbH, Germany) at − 20 °C. Subsequently, the seed sections were placed in groups on electrically conductive slides coated with indium tin oxide (ITO), and dried in a vacuum desiccator for 30 min. Desiccated sections mounted on ITO glass slides were sprayed using an HTX TM sprayer (Bruker Daltonics, Germany) with 15 mg/mL DHB, dissolved in 90%: 10% acetonitrile: water. The sprayer temperature was set to 70 °C, with a flow rate of 0.1 mL/min, and a pressure of 6 psi. 28 passes of the matrix were applied to slides with 5 s of drying time between each pass.

MALDI timsTOF MS imaging experiments were performed on a prototype Bruker timsTOF flex MS system (Bruker Daltonics, Bremen, Germany) equipped with a 10 kHz smartbeam 3D laser. Laser power was set to 80% and then fixed throughout the whole experiment. The mass spectra were acquired in positive mode, and the data were obtained over a mass range from m/z (mass-to-charge ratio) 50–1300 Da. Metabolites in the samples were identified by comparing accurate m/z values, retention times (RT), and fragmentation patterns. The imaging spatial resolution was set to 50 μm, and each spectrum consisted of 400 laser shots. MALDI-MS were normalized with the Root Mean Square, and the signal intensity in each image was shown as the normalized intensity. MS/MS fragmentations performed on the timsTOF flex MS system in the MS/MS mode were used for further detailed structural confirmation of the identified metabolites.

### Rice Panicle Blast Inoculation

The fungus (*M. oryzae* isolate Guy11) was cultured on complete medium for 2 weeks at 25 °C. The *M. oryzae* spores were collected in sterile water containing 0.2% Tween-20 and the concentration of spores was adjusted to about 1 × 10^5^ spores per mL. Rice panicles at the filling stage were completely infiltrated in the spores suspension for 3 min and sealed in a plastic bag for moisturizing, and the phenotypes were observed and recorded after 5–7 days of inoculation at 28 °C. Relative fungal growth was measured by DNA-based quantitative PCR (qPCR) using the threshold cycle value (*C*_T_) of *M. oryzae 28S rDNA* against the *C*_T_ of rice genomic *ACTIN1* DNA.

### Determination of Nutrition and Quality Indicators in Rice Seeds

The soluble sugar content of rice seeds was determined using the anthrone-sulphuric acid colorimetric method. Soluble sugar content was obtained by measuring the change of absorbance at 620 nm. The total amino acid content was determined based on the ninhydrin-ethanolic colorimetric method. The total amino acid content was obtained by measuring the change of absorbance at 570 nm. The total flavonoid content was determined by the aluminum-sodium nitrite colorimetric method. The total flavonoid content was obtained by measuring the change of absorbance at 510 nm. The amylose content was determined by using the iodine solution colorimetric method. The amylose content was obtained by measuring the change of absorbance at 620 nm. The total protein content is determined by the BCA method. The total protein content was obtained by measuring the change of absorbance at 562 nm. The free fatty content was measured by using the copper soap method. The free fatty content was obtained by determining the change of absorbance at 715 nm.

All these nutrition and quality indicators were determined using commercial kits (Cominbio Co., Ltd., Suzhou, China) according to the manufacturer's protocol. The absorbance of each reaction mixture was measured using a microplate reader (Infinite 200 Pro, Tecan, Switzerland).

## Results

### The Accumulation Pattern of Sakuranetin in Rice

**Fig. 2:**
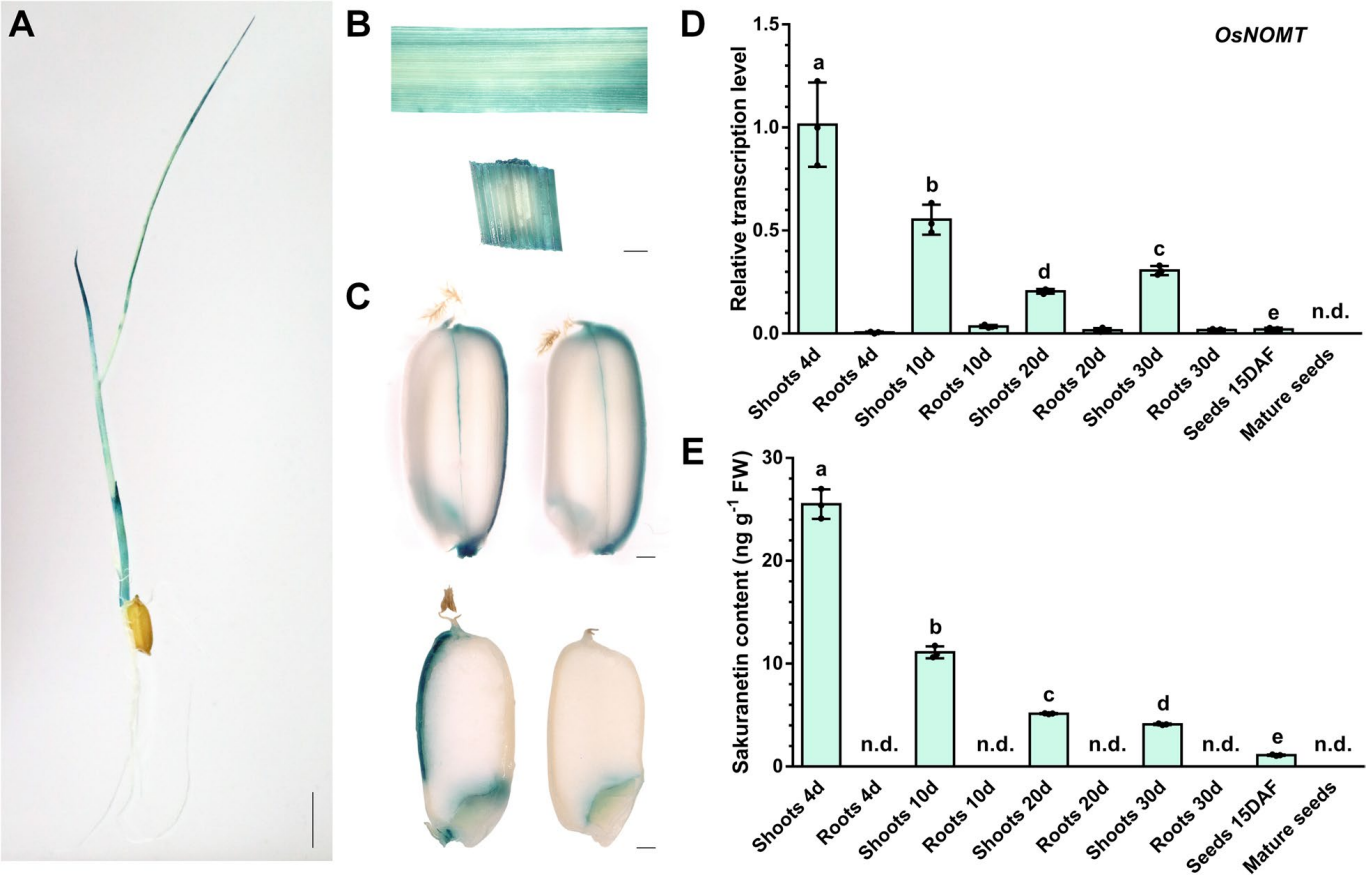
The expression pattern of *OsNOMT* and sakuranetin content in different rice tissues. **A** GUS staining of 7-day-old seedlings of *pOsNOMT::GUS*. Scale bar = 1 cm. **B** GUS staining of 20-day-old leaf (top) and leaf sheath (bottom) of *pOsNOMT::GUS*. Scale bar = 1 mm. **C** GUS staining of *pOsNOMT::GUS* seeds at 15 DAF. The top is the intact husked seeds, and the bottom is the cross section of the seeds (the left half contains the ridge of the seed, the right half does not). Scale bar = 0.5 mm. **D** qRT-PCR analysis of the expression levels of *OsNOMT* in different tissues of ZH11. **E** LC–MS/MS analysis of the sakuranetin content in different tissues of ZH11. Different letters indicate significant differences at *P* < 0.05 as determined by one-way ANOVA with Tukey’s test (mean ± s.d., n = 3, individual values and means are shown, biologically independent samples) (**D**, **E**). n.d., not detected

Previous metabolomes found that the mature rice seeds lacked sakuranetin, but presented its biosynthetic precursor, naringenin (Shi et al. 2022; Tang et al. 2022). To confirm this result, we detected the content of naringenin through the LC–MS/MS after sampling from various tissues and developmental stages. As shown in Additional file 1: Fig. S1, we found that naringenin was high in the shoots of rice seedlings, gradually increased in roots with growth and development, and was rarely present in seeds at the filling and mature stages. To investigate the accumulation pattern of the sakuranetin in rice seeds, we initially introduced the *GUS* reporter gene driven by the promoter of sakuranetin biosynthetase gene *OsNOMT* (*pOsNOMT::GUS*) into rice plants to characterize the tissue-specific expression pattern of *OsNOMT*. The GUS-staining patterns revealed that *OsNOMT* was highly expressed in the leaves and leaf sheaths of rice seedlings, and was slightly expressed in the ridges and embryos of seeds at the filling stage, with no signals in roots, husks, and endosperm (Fig. 2A–C). The quantitative real-time PCR (qRT-PCR) results also showed that *OsNOMT* was highly expressed in the shoots of rice seedlings. The expression levels decreased gradually with the growth time, while it was almost not expressed in roots and seeds (Fig. 2D). To further analyze the accumulation pattern of sakuranetin in various rice tissues, LC–MS/MS detected the content of sakuranetin. As shown in Fig. 2E, in general agreement with the *OsNOMT* expression pattern and naringenin content, sakuranetin content was high in the shoots of rice seedlings and decreasing with growth and development time, whereas it was not detected in roots. However, in contrast to naringenin, sakuranetin was less abundant than naringenin in each tissue, like previous studies, was absent in mature seeds. These results indicate that sakuranetin is absent or present in rice seeds at very low abundance.

### Engineering the Biosynthesis of Sakuranetin in the Rice Endosperm

Subsequently, we attempted to achieve accumulate sakuranetin in rice seeds through metabolic engineering and synthetic biology. First, we used the strong cauliflower mosaic virus *35S* promoter to drive *OsNOMT* (*p35S::OsNOMT-GFP*) and transform wild-type rice ZH11 (Zhonghua11) for constitutive expression in various tissues of rice. As shown in Additional file 1: Fig. S2, while *OsNOMT* was successfully expressed in rice at both transcriptional and translational levels and high levels of sakuranetin could be detected in the shoots of rice (Additional file 1: Fig. S2A–C), the effect of sakuranetin accumulation in the seeds was not satisfactory (Additional file 1: Fig. S2D).

**Fig. 3:**
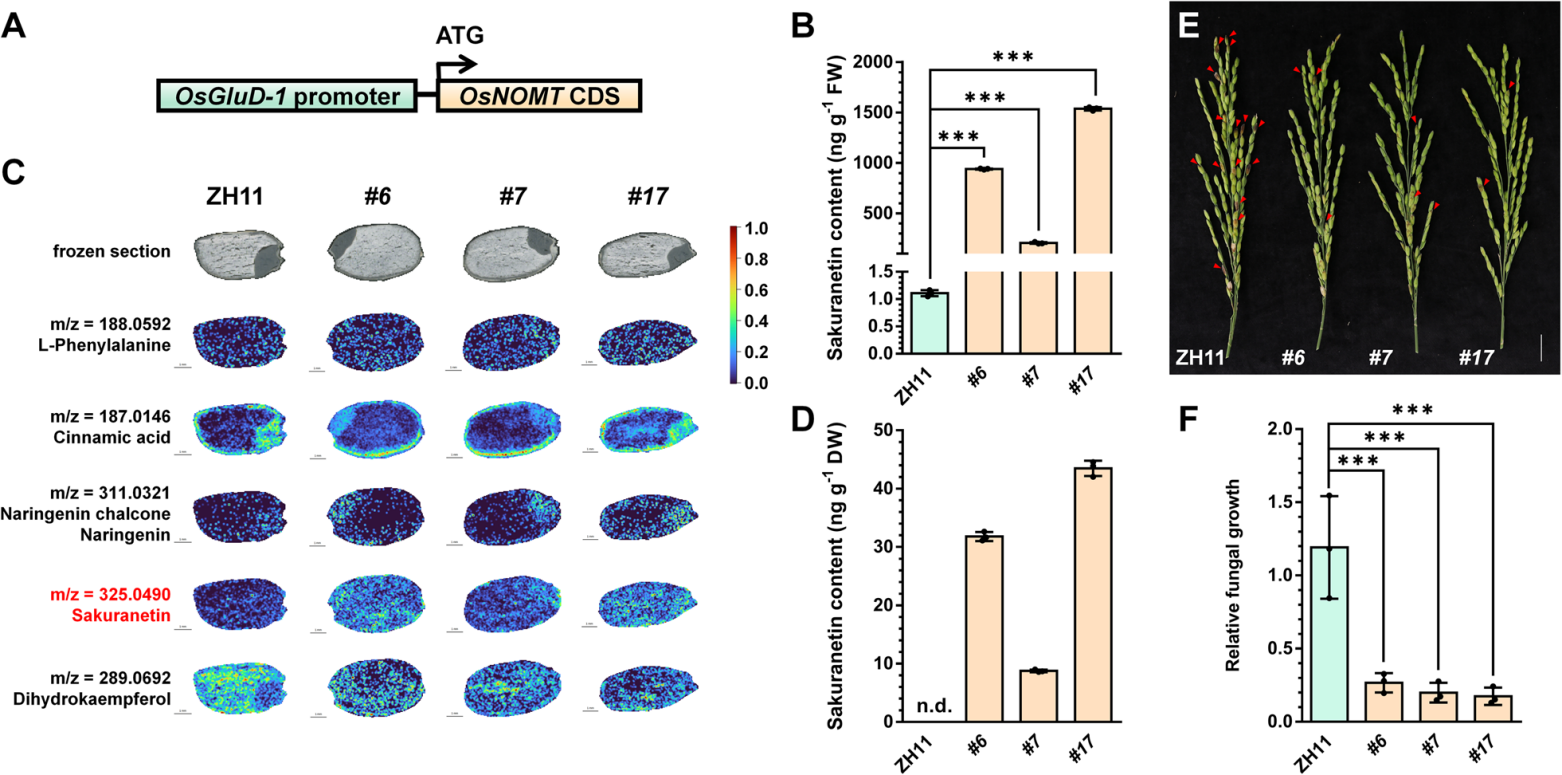
The specific expression of *OsNOMT* in endosperm resulted in the accumulation of sakuranetin and increased the blast resistance in rice seeds. **A** Schematic diagram of the *pOsGluD-1::OsNOMT* construct with the *OsNOMT* CDS, driven by the *OsGluD-1* promoter. **B** LC–MS/MS analysis of the sakuranetin content in ZH11 and *pOsGluD-1::OsNOMT* seeds at 15 DAF. *#6*, *#7*, and *#17* represent three different transgenic lines. *P*-values were determined using two-tailed Student’s *t*-tests, ***P < 0.001 (mean ± s.d., n = 3, individual values and means are shown, biologically independent samples). FW, fresh weight. **C** The MALDI-MS images of metabolites in the sakuranetin biosynthesis pathway in ZH11 and *pOsGluD-1::OsNOMT* seeds at 25 DAF. The colors in the spatial distribution images represent the relative content of metabolites, and 0 to 1 indicates sequentially increasing metabolite content. Scale bar = 1 mm. **D** LC–MS/MS analysis of the sakuranetin content in ZH11 and *pOsGluD-1::OsNOMT* seeds at the mature stage. DW, dry weight; n.d., not detected. **E** The phenotype of *pOsGluD-1::OsNOMT* panicle inoculated with rice blast. The red triangles indicate *M. oryzae-*infected rice seeds. Scale bar = 2 cm. **F** The relative fungal growth in **E**. Relative fungal growth indicates the relative fungal biomass in *M. oryzae-*inoculated panicles and was determined with fungal *28S rDNA* normalized to rice genomic *ACTIN1* DNA by DNA-based qPCR. *P*-values were determined using two-tailed Student’s *t*-tests, ***P < 0.001 (mean ± s.d., n = 3, individual values and means are shown, biologically independent samples)

Endosperm-specific SSP gene (e.g., *GluA*, *GluB*, *GluC*, *GluD*, and *Gt*) promoters have been widely utilized in plant molecular farming, where the expression levels of the *OsGluD-1* gradually increase at least until the rice seeds mature stage 30 DAF, in contrast to the gradual decrease of many other glutelin genes at later stages of seed development (Kawakatsu et al. 2008; He et al. 2011; Patti et al. 2012). Therefore, we employed 1.6 kb *OsGluD-1* promoter to drive the CDS of *OsNOMT* (*pGluD-1::OsNOMT*) to express *OsNOMT* in the rice endosperm strongly (Fig. 3A). As expected, the expression levels of *OsNOMT* (Additional file 1: Fig. S3A) and the content of sakuranetin (Fig. 3B) in rice seeds at the filling stage were found to be notably higher than wild type in three transgenic lines by qRT-PCR and LC–MS/MS. This value is higher than the sakuranetin content in the shoots of *p35S::OsNOMT-GFP* plants (Additional file 1: Fig. S2C), and even higher than sakuranetin induced by rice blast-infected in wild-type rice (Hasegawa et al. 2014). Surprisingly, the naringenin content was also increased in the *pGluD-1::OsNOMT* plants (Additional file 1: Fig. S3B), probably due to feedback regulation of the metabolic pathway. MALDI-MS imaging is a powerful technique developed in recent years for detecting the spatial distribution of metabolites and has been widely used in plant and food fields. Previous studies have used MALDI-MS imaging to reveal the distribution of different metabolites in many fruits and seeds, such as tomato fruits, strawberry fruits, *Brassica napus* seeds, and maize seeds (Feenstra et al. 2017; Lu et al. 2018; Wang et al. 2021; Li et al. 2022). To get a better insight into observing the accumulation pattern of sakuranetin in *pGluD-1::OsNOMT* seeds, we performed MALDI-MS imaging to detect the spatial distribution of relevant metabolites in the sakuranetin biosynthesis pathway in seeds at the dough stage. As shown in Fig. 3C and Additional file 1: Table S1, the levels of sakuranetin, naringenin/naringenin chalcone, and cinnamic acid were elevated in *pGluD-1::OsNOMT* plants, with unchanged levels of the synthesis initiator L-phenylalanine, and decreased levels of dihydrokaempferol. This is consistent with the previous LC–MS/MS results. Moreover, the spatial distribution of these metabolites was inconsistent. L-phenylalanine, naringenin/naringenin chalcone, and sakuranetin were almost uniformly distributed throughout the seeds, cinnamic acid was mainly distributed in the embryo and seed cortex, and dihydrokaempferol was mainly distributed in the endosperm. In the *pGluD-1::OsNOMT* plants, sakuranetin was increased throughout the seed, and dihydrokaempferol was decreased mainly in the endosperm. Notably, the spatial distribution of sakuranetin in WT is slightly different from the expression pattern of *OsNOMT* (Fig. 2C). The *OsNOMT* predominately expressed in the embryo and the dorsal side of rice seed, whereas sakuranetin accumulated throughout the seed. There are two possible reasons for this phenomenon. First, the samples of GUS staining were whole or half WT seeds, and *OsNOMT* gene expression patterns could be detected. However, the accumulation of sakuranetin in WT seeds is extremely low (Fig. 2E), and the thickness of the spatial metabolome sample section is only 20 μm, so the spatial distribution of sakuranetin cannot be accurately and meticulously detected by MALDI-MS imaging. Secondly, rice seeds may not be sliced precisely to the ridges of the seed, such as in the last image in Fig. 2C. Therefore, GUS staining and MALDI-MS imaging have different sensitivities. These two approaches complement each other, providing evidence for modifications of metabolic pathways from upstream gene levels and downstream metabolite levels, respectively.

Finally, we detected sakuranetin in rice seeds at the mature stage, and *pGluD-1::OsNOMT* plants were still much higher than the wild type. Still, it was lower than the filling stage (Fig. 3D). Considering the strong activity of sakuranetin against rice blast, the rice blast resistance of *pGluD-1::OsNOMT* panicles was determined. Once rice seeds are infected with the *M. oryzae*, the episperm will show disease spots or even turn black and die. As shown in Fig. 3E, the panicle of *pGluD-1::OsNOMT* had more seeds infected with *M. oryzae* than the wild type. Further detection of the relative fungal growth by DNA-based qPCR revealed that the *M. oryzae* biomass of transgenic panicles was much less than wild type (Fig. 3F). These suggested that the panicle blast resistance of *pGluD-1::OsNOMT* was significantly higher than that of the wild type. Generally, these results indicated that endosperm-specific expression of *OsNOMT* successfully increased the sakuranetin content and the rice blast resistance of rice seeds.

### The Nutrition and Quality of *pOsGluD-1::OsNOMT* Seeds Were not Affected

To gain insights into whether our modification to the endosperm affect rice seed nutrition and quality, we comprehensively analyzed the contents of total amino acids, soluble sugars, total flavonoids, amylose, total protein, and free fatty acids in transgenic rice and wild type.

**Fig. 4:**
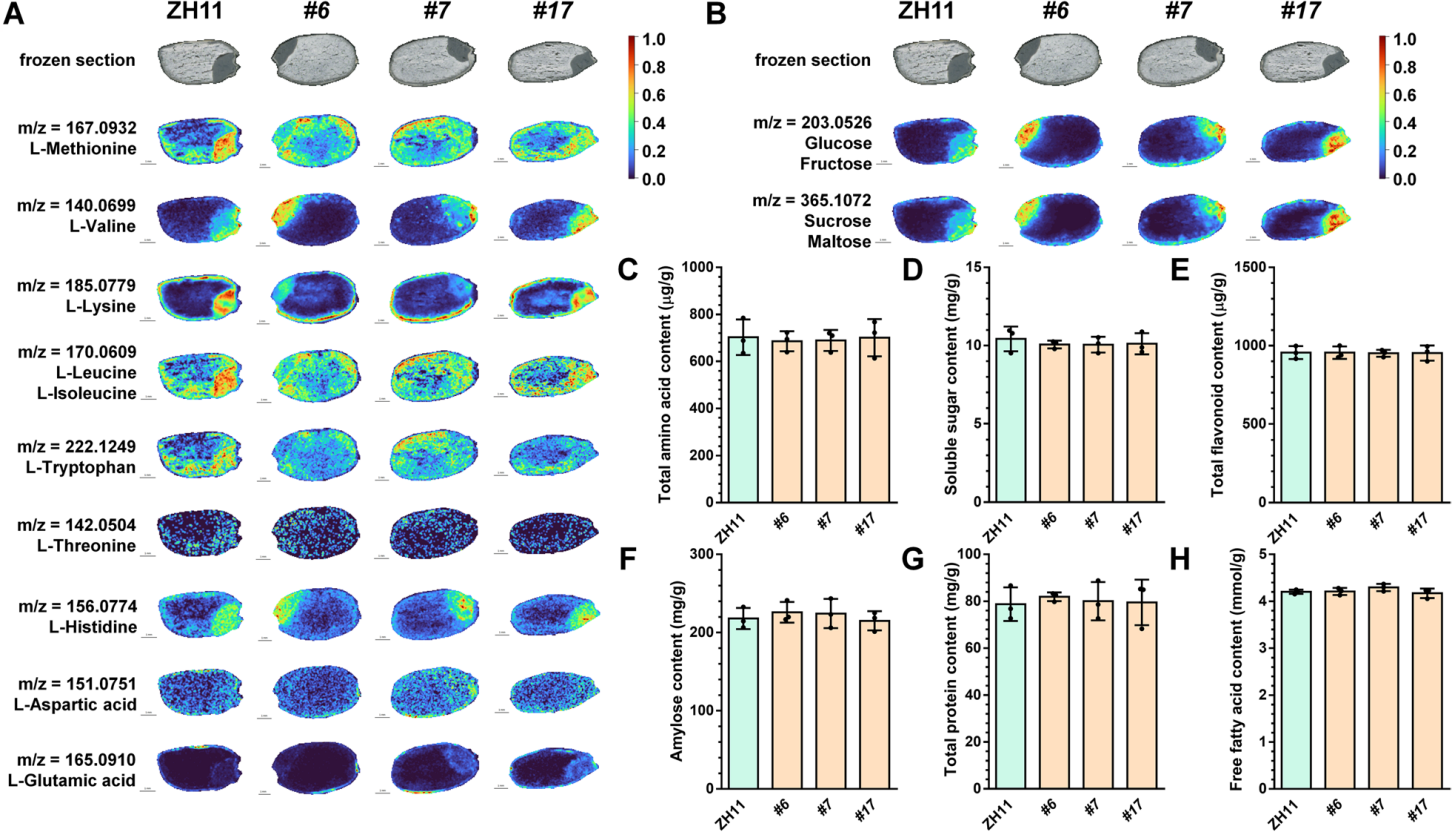
Comparison of nutrition and quality between *pOsGluD-1::OsNOMT* and wild type seeds. **A** The MALDI-MS images of the essential amino acids and amino acids that determine the eating quality in ZH11 and *pOsGluD-1::OsNOMT* seeds at 25 DAF. Scale bar = 1 mm. **B** The MALDI-MS images of of targeted free soluble sugars in ZH11 and *pOsGluD-1::OsNOMT* seeds at 25 DAF. Scale bar = 1 mm. **C**–**H** Comparison of total amino acid, soluble sugar, total flavonoid, amylose, total protein and free fatty acid content between ZH11 and *pOsGluD-1::OsNOMT* seeds at the mature stage. *P*-values were determined using two-tailed Student’s *t*-tests (mean ± s.d., n = 3, individual values and means are shown, biologically independent samples)

As the basic unit of protein, amino acids participate in not only redox homeostasis, osmoregulation, signal transduction, and other life processes but also the content of some amino acids such as aspartic acid and glutamic acid in rice grains, can affect the eating quality (Matsuzaki et al. 1992; Kelly and Pearce 2020; Guo et al. 2021). The MALDI-MS imaging detected 9 essential amino acids (L-methionine, L-isoleucine, L-leucine, L-valine, L-lysine, L-phenylalanine, L-tryptophan, L-threonine, and L-histidine) and 2 amino acids that determine the eating quality (L-aspartic acid and L-glutamic acid) of rice seeds at the dough stage (Figs. 3C, 4A and Additional file 1: Table S1). It is found that the L-histidine content in *pOsGluD-1::OsNOMT* seeds increased, and the main increasing segmentation was the embryo, while the content of L-methionine and L-isoleucine/L-leucine slightly decreased. Their distribution fell in the embryo and raised in the endosperm. The contents and spatial distribution of other amino acids did not change significantly. Furthermore, the content of total amino acids in the mature seeds of transgenic plants showed no significant difference from the wild type, suggesting that changes in the content and spatial distribution of a few amino acids did not affect the content of total amino acids (Fig. 4C). Soluble sugars are important energy storage substances and the main source of sweetness in plant seeds. Their content is closely related to the nutrition and quality of seeds (Zhu et al. 2007). As shown in Fig. 4B and Additional file 1: Table S1, the detection of the 4 main soluble sugars (glucose, fructose, sucrose, and maltose) by MALDI-MS imaging revealed that the soluble sugar content was slightly elevated in *pOsGluD-1::OsNOMT* seeds, and the main segmentation of the elevation was in the embryo. We also analyzed the total soluble sugar content of transgenic plant seeds at the mature stage and observed no significant difference compared to the wild type, which may be due to the other soluble sugars in the rice seeds (Fig. 4D). In addition to amino acids and soluble sugars, the content of flavonoids, amylose, protein, and free fatty acids in seeds is also a crucial factor in determining its nutrition, taste and quality (Birla et al. 2017; Kasote et al. 2022). The contents of total flavonoid, amylose, total protein and free fatty acid in the mature seeds were detected, and there was no significant difference between *pOsGluD-1::OsNOMT* plants and wild type (Fig. 4E–H). In summary, these results show that the nutrition and quality of *pOsGluD-1::OsNOMT* seeds were not affected.

### The Growth and Development of *pOsGluD-1::OsNOMT* Plants Were not Affected

**Fig. 5:**
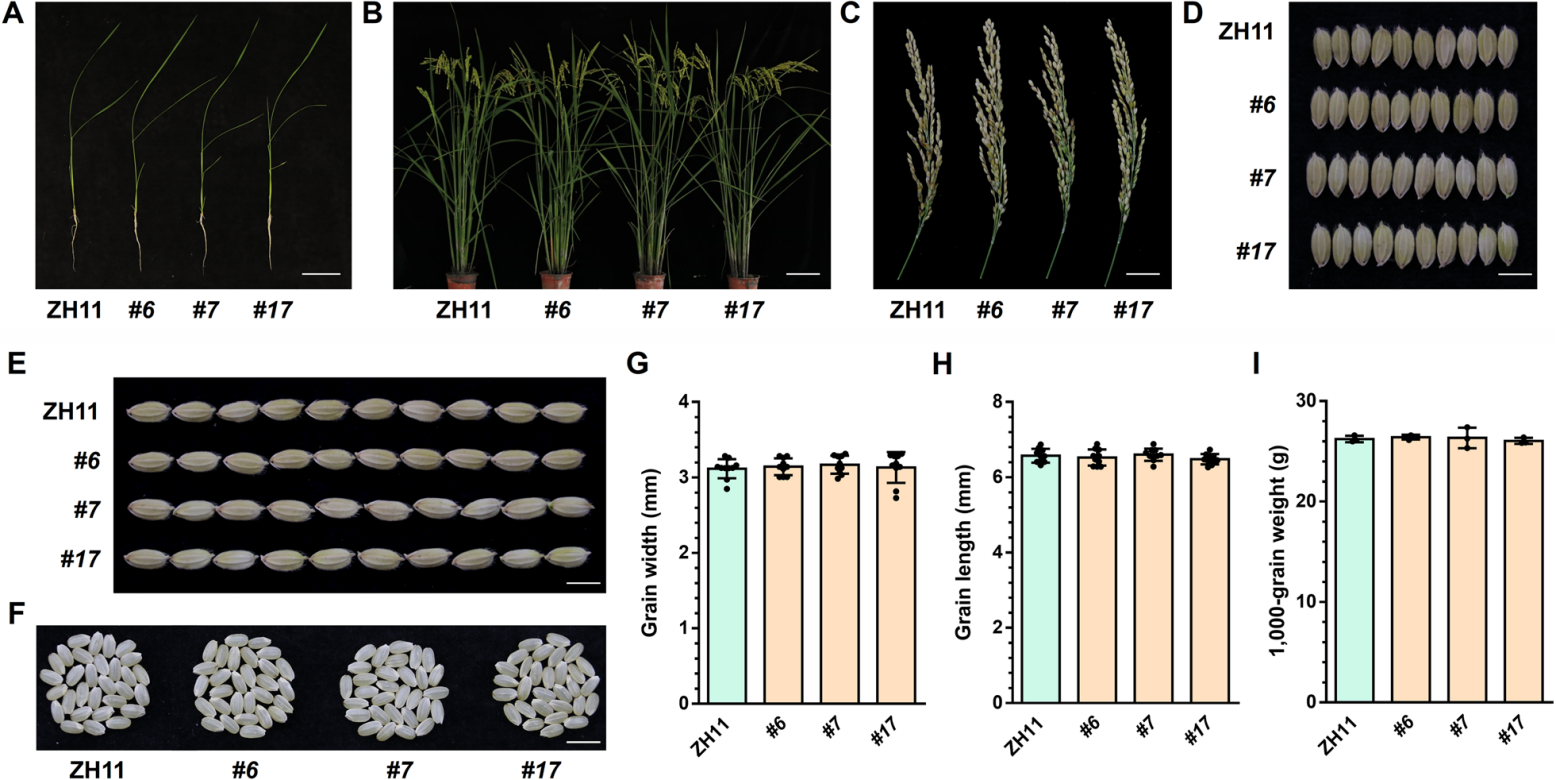
The phenotype of *pOsGluD-1::OsNOMT*. **A** Phenotype of 14-day-old ZH11 and *pOsGluD-1::OsNOMT* seedlings. Scale bar = 5 cm. **B** Phenotypes of ZH11 and *pOsGluD-1::OsNOMT* at the reproductive stage. Scale bar = 15 cm. **C** The phenotypes of panicles at the grain maturation stage detached from the ZH11 and *pOsGluD-1::OsNOMT.* Scale bar = 3 cm. **D**, **E** Mature grains of ZH11 and *pOsGluD-1::OsNOMT.* Scale bar = 0.5 cm. **F** Husked grains of ZH11 and *pOsGluD-1::OsNOMT.* Scale bar = 0.8 cm. **G**–**I** Comparison of grain width (n = 10), grain length (n = 10), and 1000-grain weight (n = 3) between ZH11 and *pOsGluD-1::OsNOMT. P* values were determined using two-tailed Student’s *t*-tests (mean ± s.d., individual values and means are shown, biologically independent samples)

Apparently, the use of metabolic engineering to increase the biosynthesis of sakuranetin in endosperm caused changes in the content of some metabolites (Figs. 3C, 4A, B). Next, we explored whether this modification affected the growth and development of *pOsGluD-1::OsNOMT* plants. We tracked and observed the phenotypes of *pOsGluD-1::OsNOMT* plants at different growth stages, and found that the plant architecture, panicle morphology, and maturity stage were the same as the wild type (Fig. 5A–C). As a key agricultural trait, grain size determines grain yield and quality in rice (Zuo and Li 2014). The grain size of *pOsGluD-1::OsNOMT* plants was recorded and analyzed in focus, and there were no significant differences between the three transgenic lines and the wild type in terms of grain width (Fig. 5D, G) and grain length (Fig. 5E, H). The threshed seeds also showed the same phenotype as the wild type (Fig. 5F). In addition, we measured the 1,000-grain weight of transgenic plants, and there was no significant difference between *pOsGluD-1::OsNOMT* plants and wild type (Fig. 5I). Altogether, these results demonstrated that the growth and development of *pOsGluD-1::OsNOMT* were not affected. In fact, based on our observations in the phytotron and the field, we also found the vegetative and reproductive phenotypes of *p35S::OsNOMT-GFP* were not significantly different from the WT at all stages of growth and development. This suggested that the accumulation of sakuranetin in various tissues of rice does not influence its growth and development.

### The Proposed Work Model of *pOsGluD-1::OsNOMT*

To investigate whether this work is generalizable to other cereal crops, we examined naringenin and sakuranetin content in maize (*Zea mays*), sorghum (*Sorghum bicolor*), millet (*Setaria italica*), oat (*Avena sativa*), and coix (*Semen coicis*). As shown in Additional file 1: Fig. S4, naringenin and sakuranetin were much higher in maize and sorghum than in the other grains, while the naringenin and sakuranetin contents in millet, oat, and coix were essentially similar to the rice. Overall, these cereal seeds lacked sakuranetin or contained very little sakuranetin. Thus, our strategy of metabolic engineering to accumulate sakuranetin in endosperm can be extended to other cereal crops.

**Fig. 6:**
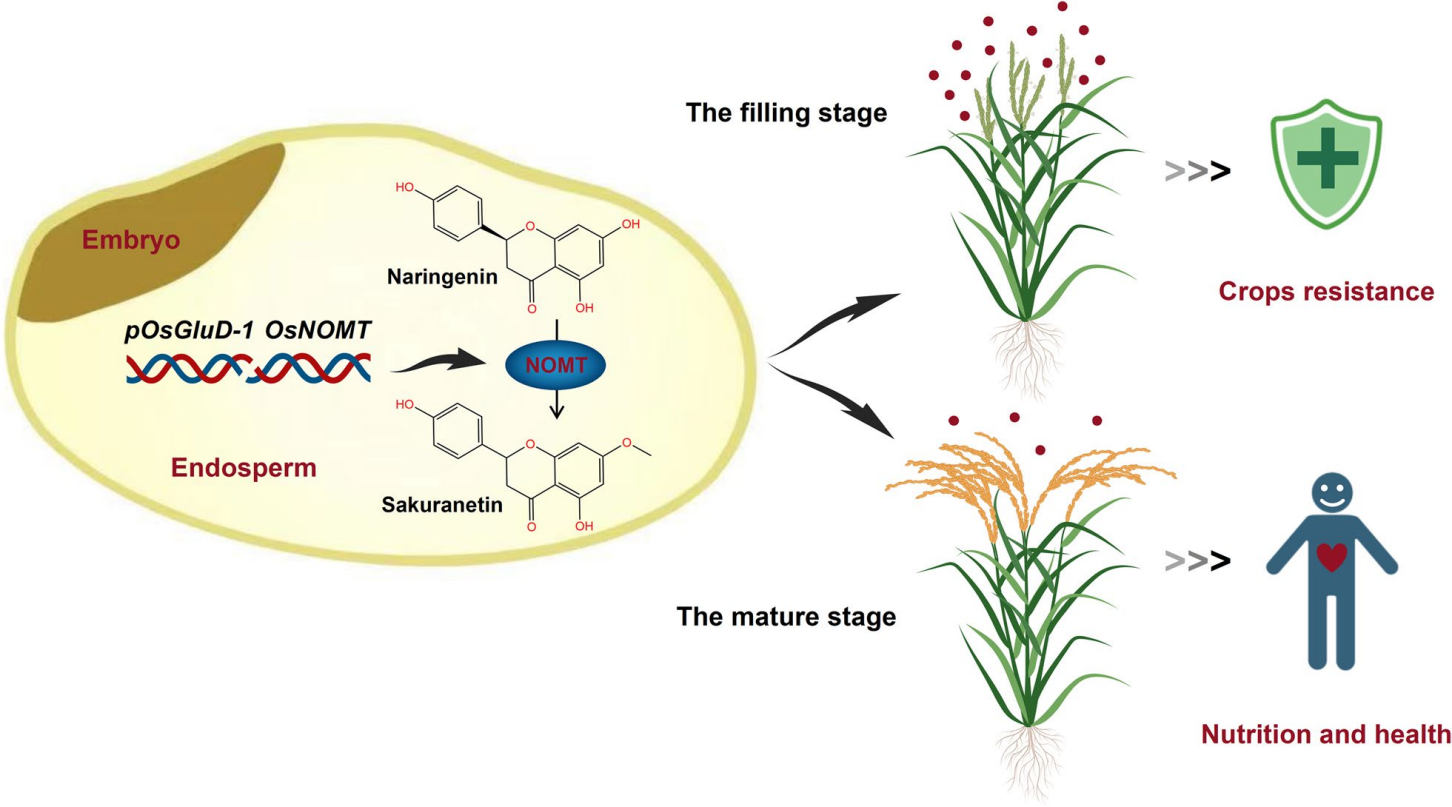
The proposed work model illustrating how endosperm-specific expression of *OsNOMT* leads to the accumulation of sakuranetin in the rice seeds. The expression of the sakuranetin biosynthesis gene *OsNOMT* was driven by the promoter of the endosperm-specific glutelin gene *OsGluD-1*, and OsNOMT successfully catalyzes naringenin synthesis of sakuranetin in the rice seeds. Sakuranetin content was highest at the filling stage and decreased with seed maturation. During the filling stage, the rapid and massive accumulation of sakuranetin is proposed to improve disease resistance in crop panicles by acting as a phytoalexin. At the mature stage, the lower abundance of sakuranetin confer potential nutritional and health benefits to humans. The dark brown color in the proposed model represents the rice seed embryo, the yellow color represents the cortex (rice bran layer), the beige color represents the endosperm, and the small red dots represent sakuranetin

Based on all the above results, a working model of *pOsGluD-1::OsNOMT* in rice seeds was proposed (Fig. 6). Specifically, the expression of the sakuranetin biosynthesis gene *OsNOMT* was driven by the promoter of the endosperm-specific glutelin gene *OsGluD-1*, resulting in the successful accumulation of sakuranetin in rice seeds. Sakuranetin content was highest at the filling stage and decreased with seed maturation. During the filling stage, the rapid and massive accumulation of sakuranetin is proposed to improve disease resistance in crop panicles by acting as a phytoalexin. At the mature stage, the lower abundance of sakuranetin confers potential nutritional and health benefits to humans. It should be noted that this modification does not affect the nutrition and yield of rice. Therefore, our study represents an entirely new paradigm for generating biofortified crops through metabolic engineering and synthetic biology, a win–win strategy for both plants and humans.

## Discussion

Plants have evolved a variety of defense mechanisms against pathogen attack, such as strengthening local cell walls, regulating phytohormone signals, activating plant innate immune response, and synthesizing phytoalexins (López et al. 2008; Piasecka et al. 2015; Hacquard et al. 2017). Rice phytoalexins include diterpenoids, phenylamides, and flavonoids, of which sakuranetin is the only flavonoid phytoalexin (Valletta et al. 2023). In fact, compared with sakuranetin and uncommon phenylamides, the research on diterpenoid phytoalexins is more extensive and in-depth. However, there are numerous species of diterpenoids phytoalexin, and the synthetic pathway is multistep and complex (Schmelz et al. 2014; Morelli and Rodriguez-Concepcion 2023). Moreover, the enzymes involved in the synthesis of diterpenoids phytoalexin have different catalytic mechanisms, different tissue specificity in plant distribution, and different suborganelle localization in the plastid, which increases the difficulty of modifying their metabolic pathways (Lange and Ghassemian 2003). In addition to phytoalexin, glucosinolates (GSLs) are another well-known class of secondary defense metabolites produced by plants, which mainly accumulate in the seeds of Brassicales plants (Grubb and Abel 2006; Mitreiter and Gigolashvili 2021). However, being an anti-nutritional factor, it can significantly impinge on the taste and nutritional value of the plant's edible tissues. As a result, the use of metabolic engineering strategies aimed at enriching rice seeds with glucosinolates would not be considered an optimal choice either (Xu et al. 2023). In recent years, the strong activity against rice leaf blast of sakuranetin has been revealed by many studies (Kodama et al. 1992; Hasegawa et al. 2014; Murata et al. 2020). Relatively, with a simple structure, clear synthetic steps, a lack of odor, and diverse physiological activities, sakuranetin presents a promising solution for balancing resistance, growth, nutrition, and quality in rice. Seed is an important organ of rice that provides energy for plant growth and nutrients for humans. Nevertheless, sakuranetin is deficient in the rice grains (Fig. 2E). In this study, the sakuranetin-rich biofortified rice was generated by modifying the biosynthesis pathway of rice endosperm. As expected, the panicle blast resistance of the transgene rice was significantly higher than WT (Fig. 3E, F).

It is widely recognized that the metabolites in plants are characterized by species specificity and spatio-temporal specificity, meaning that certain metabolic processes and enzymes responsible for catalyzing these metabolic processes exhibit the same properties (Hanson and Gregory 2002; De-la-Cruz Chacón et al. 2013). The isoenzymes distributed in various tissues may give rise to different catalytic properties and regulatory mechanisms. Furthermore, the substrate levels, coenzymes, and other factors related to enzyme activity or regulatory mechanisms are also diverse in various tissues. Therefore, modifying of a specific metabolic pathway in one plant is often challenging to apply to another species. For instance, the activities of dihydrofavonol 4-reductase (DFR) and anthocyanidin synthase (ANS) are required in tissues such as leaves, fruits, and corollas of many dicot plants that naturally synthesize anthocyanins (Wang et al. 2022). In contrast, the endosperm of the monocot plants rice is inherently deficient in anthocyanins, which complicates the modification of their synthesis pathway in the rice endosperm (Zhu et al. 2017). Unlike anthocyanins, sakuranetin, which is also flavonoid, is naturally present in rice, even though its content and the expression of the synthase gene *OsNOMT* vary in different tissues and developmental stages of rice (Fig. 2D, E). We achieved efficient and massive biosynthesis of sakuranetin in the rice endosperm by introducing a single-step metabolic pathway using rice endogenous promoters, synthesis genes and substrates. A precedent exists for utilizing species' endogenous advantages to produce biofortified crops easily and efficiently. Li et al. knocked out the enzyme gene that catalyzes 7-DHC (a precursor for provitamin D_3_ biosynthesis) biosynthesis of cholesterol in Solanaceae plants by genome editing and produced a provitamin D_3_-rich biofortified plant by modifying a single-step metabolic pathway (Li et al. 2022).

Besides being essential for plants to resist various stresses, sakuranetin has many health-promoting effects (Stompor 2020; Junaid et al. 2022). For example, sakuranetin exhibits anti-tumor, anti-viral, anti-parasitic, anti-inflammatory, and antioxidant physiological activities. Previous studies have found that sakuranetin demonstrates anti-proliferative properties against ESCC, B16BL6 melanoma, and Colo 320 (Ugocsai et al. 2005; Hong and Ying 2015; Drira and Sakamoto 2016). Sakuranetin was also reported to have antiviral activity against human rhinovirus 3 and influenza B virus (Choi 2017; Kwon et al. 2018). In addition, sakuranetin attenuated chronic allergic airway inflammation in mice by inhibiting MAPK and STAT3-SOCS3 (Santana et al. 2019). Rice is a vital food crop with a long history of cultivation and consumption. It provides more than 20% of the calories for more than half of the world's population and up to 50% of the calories for the population of Asia (Gutaker et al. 2020; Sapwarobol et al. 2021). Consequently, the endogenous synthesis of sakuranetin in rice seeds is of great significance to human health. Biofortified rice can not only supply people with sakuranetin conveniently and directly in their daily diet. Still, it can also be developed and processed into healthcare products to satisfy a broader consumption range.

In *pOsGluD-1::OsNOMT* plants, the sakuranetin levels were observed to plummet as the rice reached maturity (Fig. 3B, D). According to Kawakatsu et al., the expression levels of *OsGluD-1* remained high in the late stage of seed development (Kawakatsu et al. 2008), which ruled out the possibility that the work efficiency of *pOsGluD-1::OsNOMT* dropped sharply at the mature stage. The substrate naringenin content was reduced in seeds at the mature stage (Additional file 1: Fig. S1), which may partially explain the reduced sakuranetin content. Ogo et al. once specifically co-expressed *OsPAL* and *OsCHS* by *OsGluB-1* promoter in rice endosperm to achieve naringenin accumulation (Ogo et al. 2013). Therefore, simultaneously specifically expressing flavonoid synthase genes upstream of *OsNOMT* in the endosperm to generate a sufficient supply of naringenin could result in higher levels of sakuranetin in rice seeds. We utilized *OsGluD-1*, which is more persistently expressed than *OsGluB-1* in rice seeds, as an endosperm-specific promoter to achieve sakuranetin accumulation in seeds efficiently. Sakuranetin not only has the nutritional function of naringenin and other flavonoids but is also unique for its brilliant resistance to phytopathogen.

Moreover, based on our detection in various cereal crops, naringenin is prevalent in the grain. At the same time, sakuranetin is deficient or insufficient (Additional file 1: Fig. S4), suggesting that our modification strategy has the potential to be applied to other cereal crops. In recent years, the emerging MALDI-MS imaging technique has been used to investigate the dynamic changes of amino acids during germination of rice seeds and the spatial distribution of lipids in mature rice seeds (Ren et al. 2023; Zhang et al. 2023). With the MALDI-MS imaging, we not only revealed the spatial distribution of sakuranetin for the first time but also evaluated the spatial distribution of multiple nutrients in the seeds of *pOsGluD-1::OsNOMT* plants (Figs. 3C, 4A, B). Before this study, HPLC and DPBA (diphenyl boric acid 2-amino ethyl ester) staining were commonly used to detect flavonoids in biofortified rice seeds (Ogo et al. 2013, 2016). Herein, more specifical, precise, and comprehensive observation on the spatial distribution of multiple metabolites in different segmentations of rice seeds provides a new assessment method for detecting the effects of metabolic engineering.

## Conclusion

Collectively, we generated a sakuranetin-rich biofortified rice by metabolic engineering in which the expression of the sakuranetin biosynthesis gene *OsNOMT* was driven by the promoter of the endosperm-specific glutelin gene *OsGluD-1*, resulting in the successful accumulation of sakuranetin in rice seeds. The seeds of *pOsGluD-1::OsNOMT* plants may combine both resistance and nutritional dual functions, and this modification of the metabolic pathway did not affect plant nutrition and growth. This innovative attempt at engineering sakuranetin-rich endosperm rice would motivate the nutrient fortification of cereal crops.

### Supplementary Information

**Additional file 1. Fig. S1.** LC-MS/MS analysis of the naringenin content in different tissues of ZH11. **Fig. S2.** The sakuranetin content in *p35S::OsNOMT-GFP*. **Fig. S3.** The expression level of *OsNOMT* and naringenin content in *pOsGluD-1::OsNOMT* seeds at the filling stage. **Fig. S4.** The naringenin content and sakuranetin content in different cereal crops. **Table S1.** The relative quantification of selected metabolites by MALDI-MS imaging. **Table S2.** Primers list.

## Data Availability

The datasets used and/or analyzed during the current study are available from the corresponding author on reasonable request.

## Abbreviations

NOMT: Naringenin 7-*O*-methyltransferase
LC–MS/MS: Liquid chromatography tandem mass spectrometry
MALDI-MS: Matrix-assisted laser desorption/ionization mass spectrometry
BPH: Brown planthopper
ESCC: Esophageal squamous cell carcinoma
Colo 320: Colon cancer
PAL: Phenylalanine ammonialyase
C4H: Cinnamic acid 4-hydrolase
4CL: Coumarin-CoA ligase
CHS: Chalcone synthase
CHI: Isomerase
SSPs: Seed storage proteins
DAF: Days after flowering
ZH11: Zhonghua11
GSLs: Glucosinolates
DFR: Dihydrofavonol 4-reductase
ANS: Anthocyanidin synthase
DPBA: Diphenyl boric acid 2-amino ethyl ester
