# Supplementary material for: Biofortified Rice Provides Rich Sakuranetin in Endosperm
Source: Rice (N Y). 2024 Mar 2;17:19. doi: 10.1186/s12284-024-00697-w (PMC10908774; doi:10.1186/s12284-024-00697-w)

**
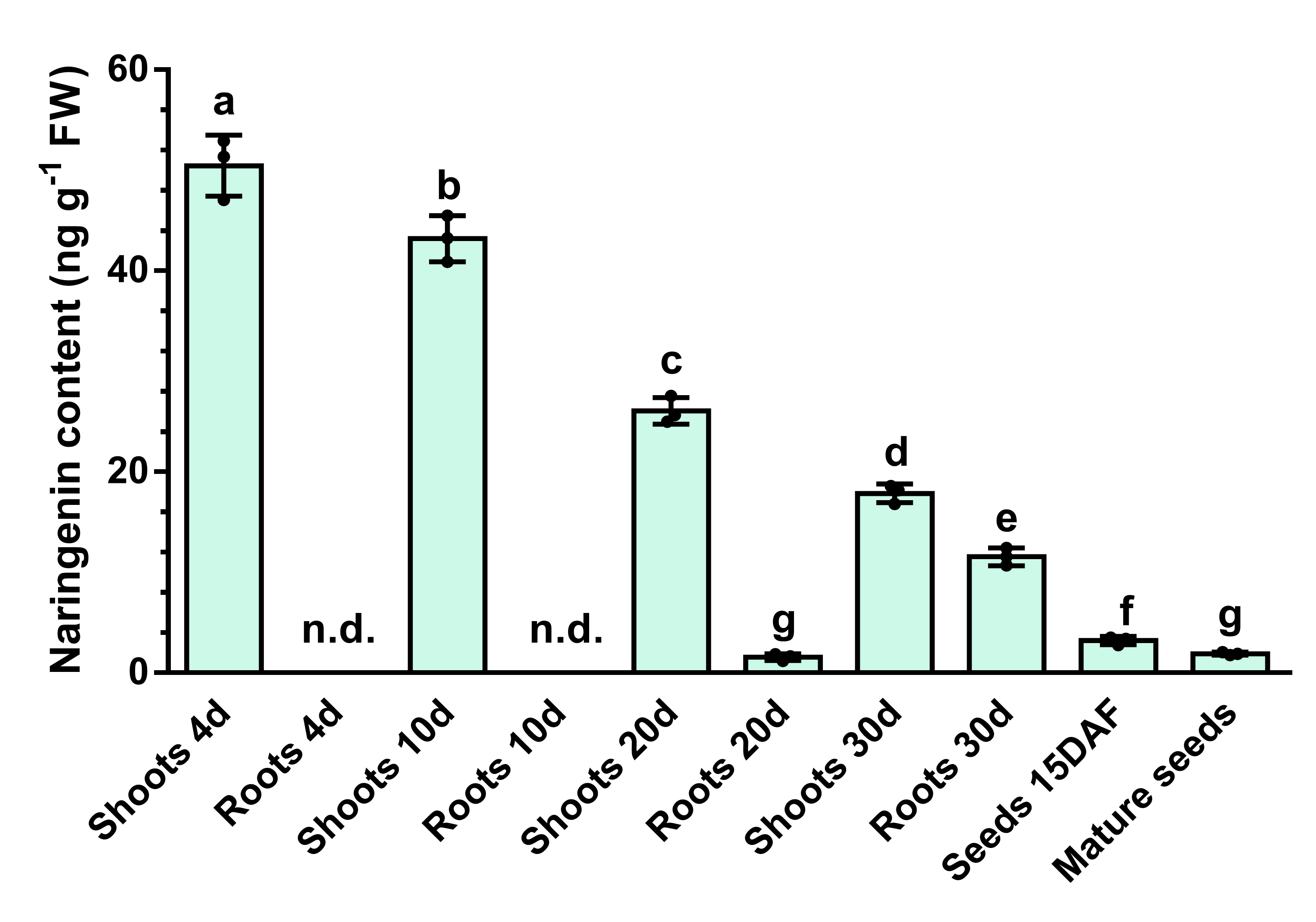
**

**Fig. S1** [LC-MS/MS](http://kns.cnki.net/KCMS/detail/detail.aspx?dbcode=CJFQ&dbname=CJFDPREP&filename=GWSQ201822003&v=MDg2ODBGckNVUkxLZVp1Um9GaURtVkxyTklqcllmN0c0SDluT3JZOUZaNFI4ZVgxTHV4WVM3RGgxVDNxVHJXTTE=) analysis of the naringenin content in different tissues of ZH11. Different letters indicate significant differences at *P* < 0.05 as determined by one-way ANOVA with Tukey’s test (mean ± s.d., n = 3, individual values and means are shown, biologically independent samples). FW, fresh weight. n.d., not detected.


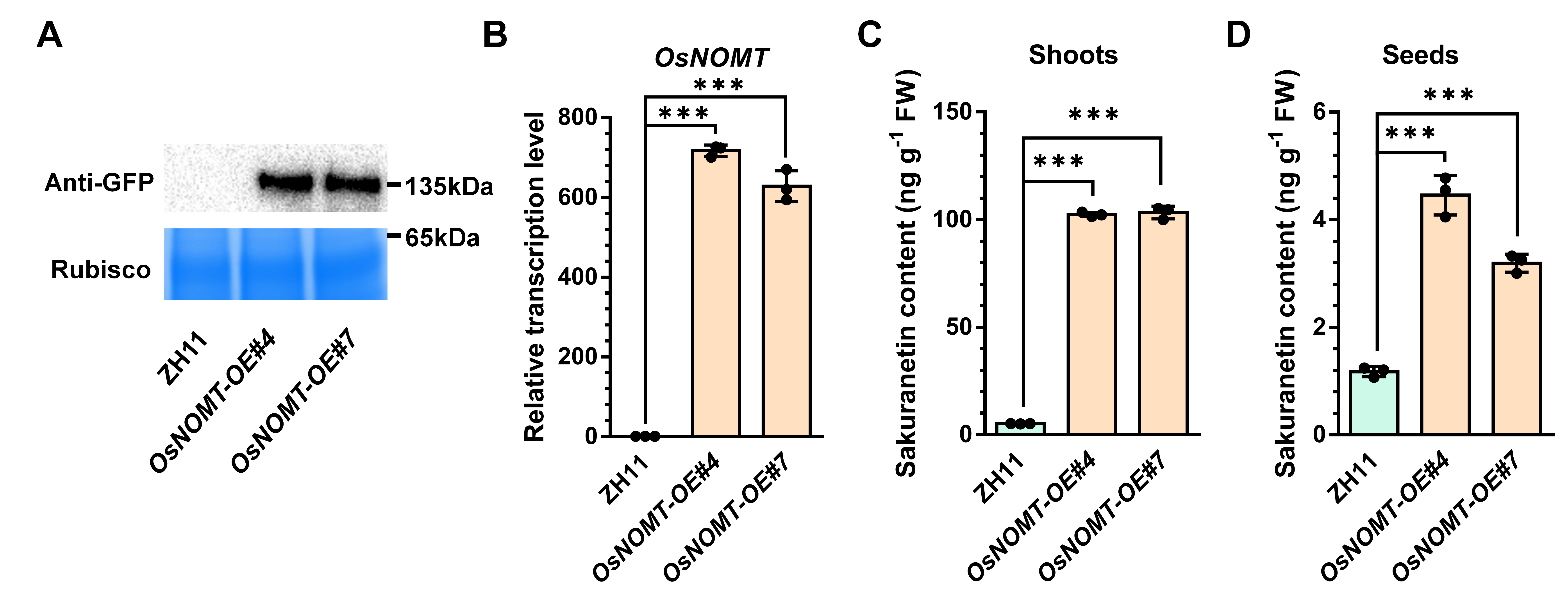


**Fig.** **S2** The sakuranetin content in *p35S::OsNOMT-GFP*. **A** Western blot analysis the protein levels of OsNOMT-GFP in 7-day-old shoots of *p35S::OsNOMT-GFP*. *OsNOMT-OE#4* and *OsNOMT-OE#7* represent two different transgenic lines. **B** qRT-PCR analysis of the expression levels of *OsNOMT* in 7-day-old shoots of *p35S::OsNOMT-GFP*. **C**, **D** [LC-MS/MS](http://kns.cnki.net/KCMS/detail/detail.aspx?dbcode=CJFQ&dbname=CJFDPREP&filename=GWSQ201822003&v=MDg2ODBGckNVUkxLZVp1Um9GaURtVkxyTklqcllmN0c0SDluT3JZOUZaNFI4ZVgxTHV4WVM3RGgxVDNxVHJXTTE=) analysis of the sakuranetin content in 7-day-old shoots and 15 DAF seeds of *p35S::OsNOMT-GFP*. *P*-values were determined using two-tailed Student’s *t*-tests, *** *P* < 0.001 (mean ± s.d., n = 3, individual values and means are shown, biologically independent samples) (**B**–**D**). FW, fresh weight.

**
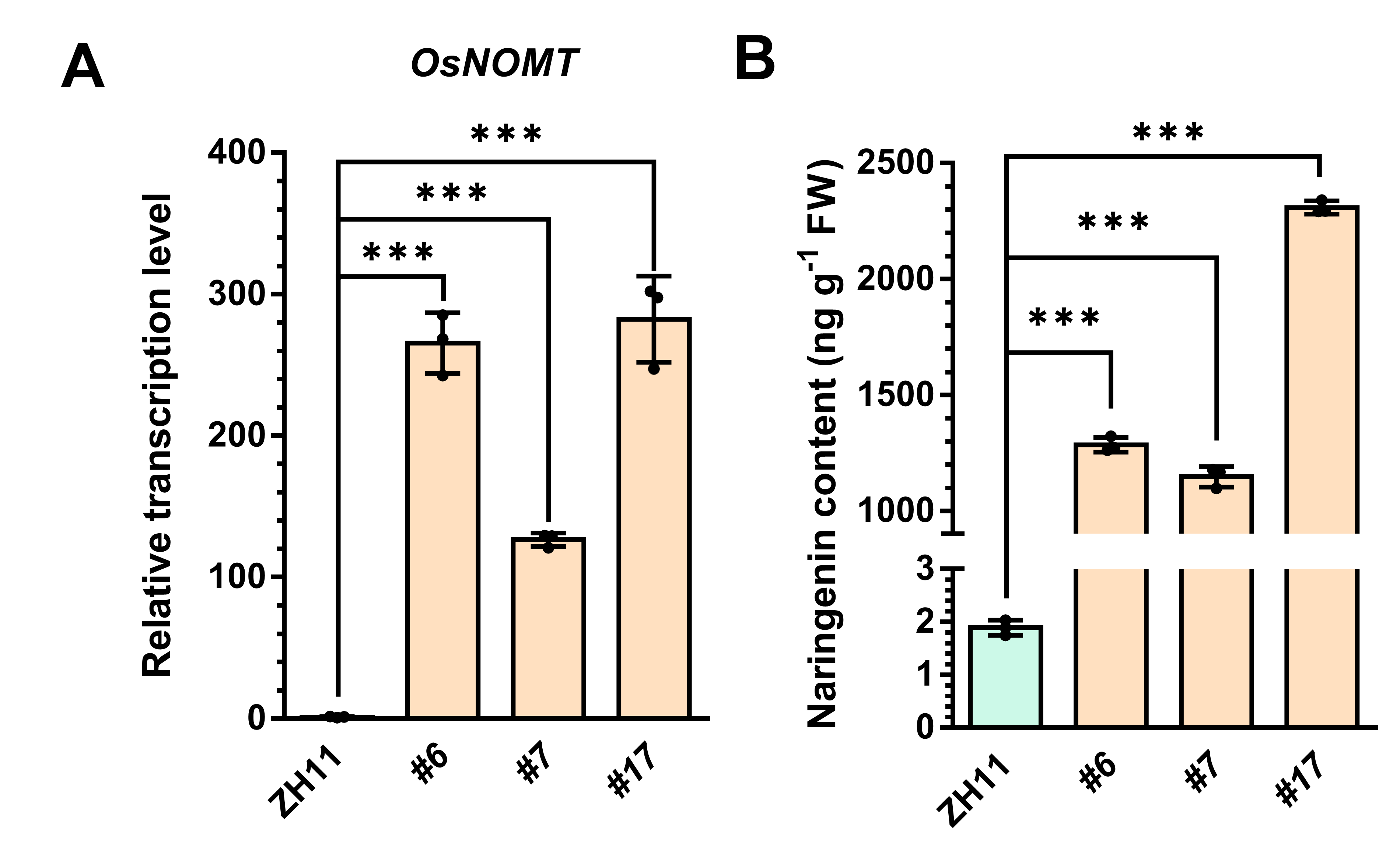
**

**Fig. S3 A**, **B** The expression level of *OsNOMT* and naringenin content in *pOsGluD-1::OsNOMT* seeds at the filling stage. *P*-values were determined using two-tailed Student’s *t*-tests, *** *P* < 0.001 (mean ± s.d., n = 3, individual values and means are shown, biologically independent samples). FW, fresh weight.


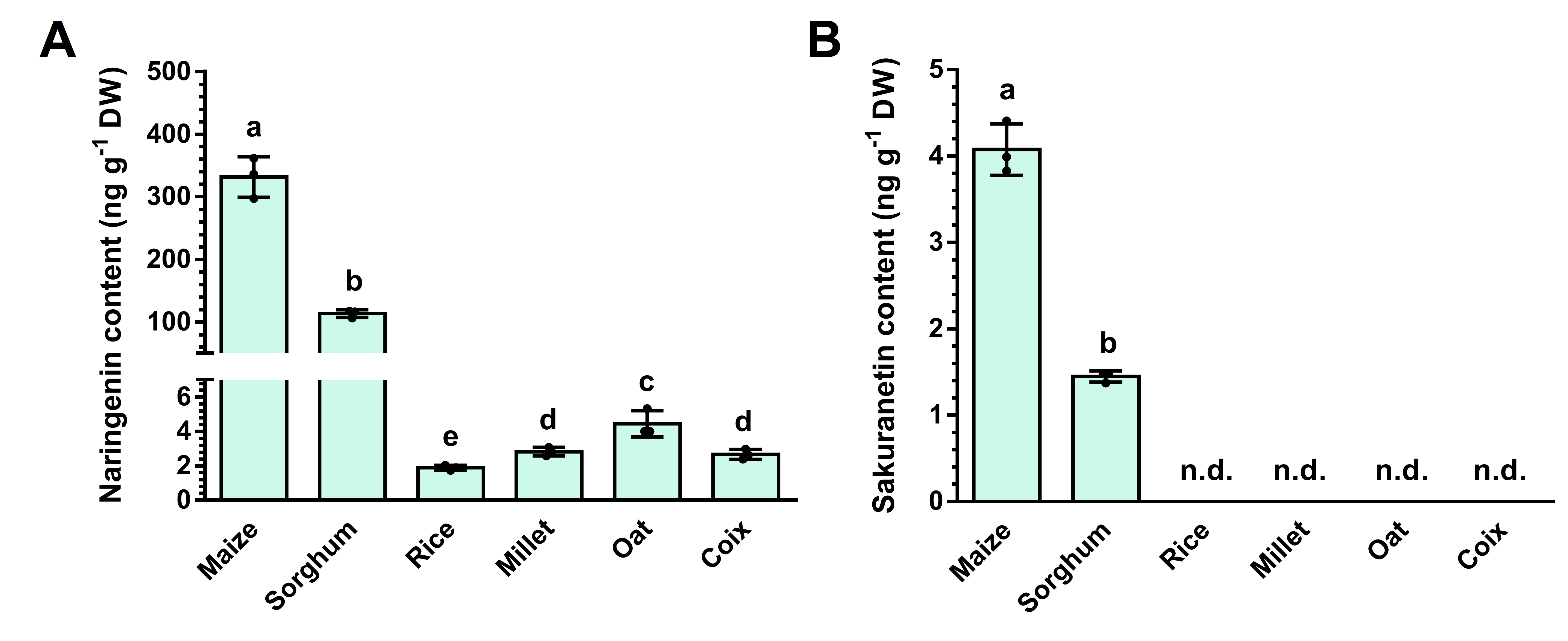


**Fig. S4 A**, **B** The naringenin content and sakuranetin content in different cereal crops. Different letters indicate significant differences at *P* < 0.05 as determined by one-way ANOVA with Tukey’s test (mean ± s.d., n = 3, individual values and means are shown, biologically independent samples). DW, dry weight. n.d., not detected.

**Table S1.** The relative quantification of selected metabolites by MALDI-MS imaging.


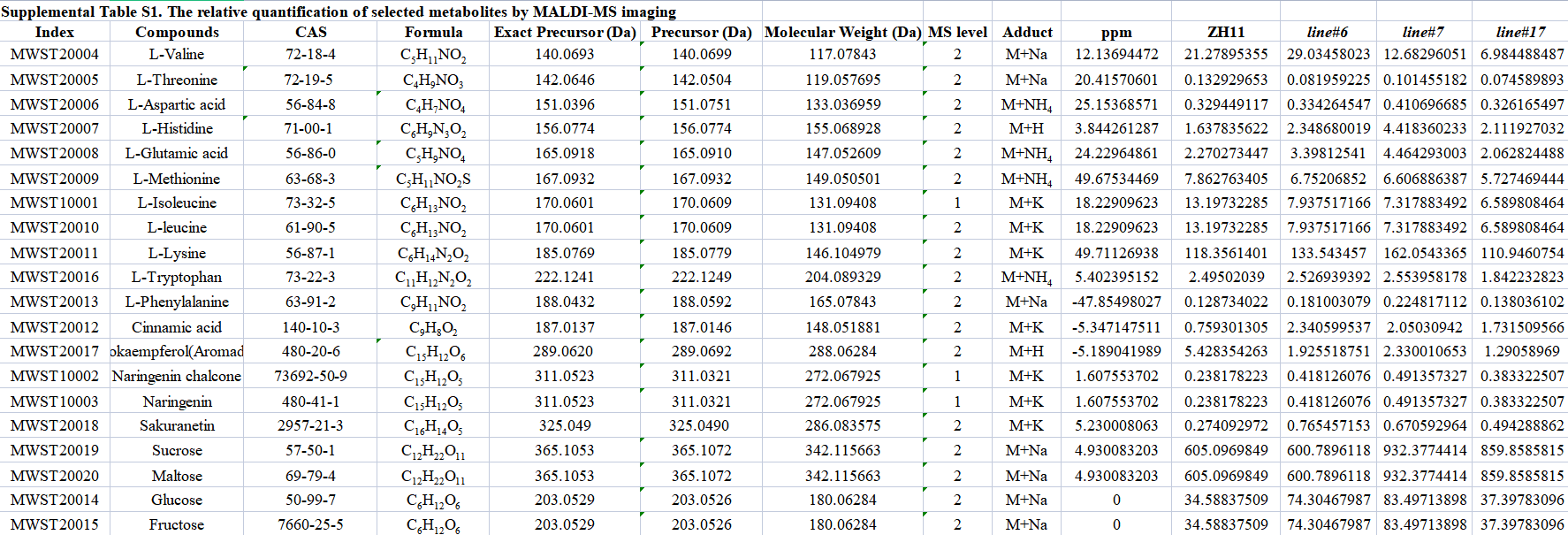


**Table S2.** Primers list.


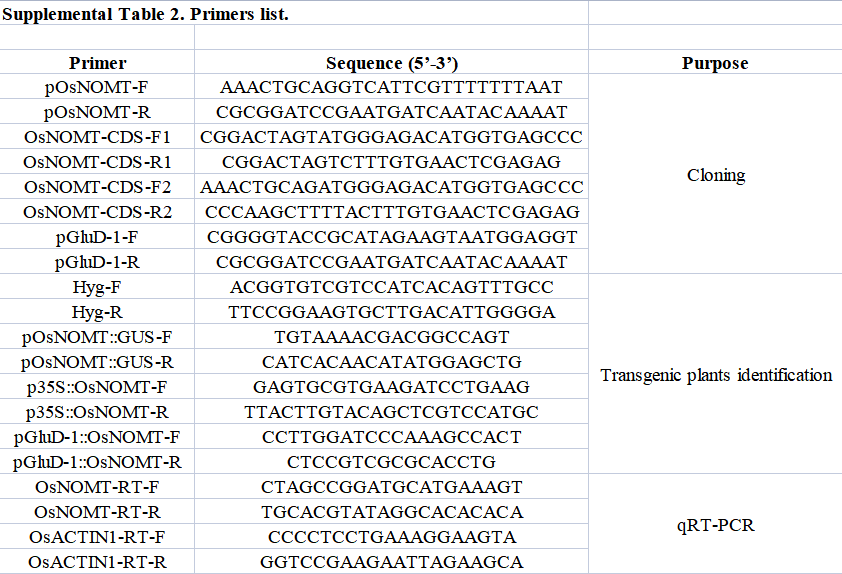

Supplement: Supplementary file 1 — Additional file 1. Fig. S1. LC-MS/MS analysis of the naringenin content in different tissues of ZH11. Fig. S2. The sakuranetin content in p35S::OsNOMT-GFP. Fig. S3. The expression level of OsNOMT and naringenin content in pOsGluD-1::OsNOMT seeds at the filling stage. Fig. S4. The naringenin content and sakuranetin content in different cereal crops. Table S1. The relative quantification of selected metabolites by MALDI-MS imaging. Table S2. Primers list. [file 12284_2024_697_MOESM1_ESM.docx]
